# Intravitreal TSG-6 suppresses laser-induced choroidal neovascularization by inhibiting CCR2^+^ monocyte recruitment

**DOI:** 10.1038/srep11872

**Published:** 2015-07-07

**Authors:** Sang Jin Kim, Hyun Ju Lee, Ji-Hyun Yun, Jung Hwa Ko, Da Ye Choi, Joo Youn Oh

**Affiliations:** 1 Department of Ophthalmology, Samsung Medical Center, Sungkyunkwan University School of Medicine, 50 Irwon-dong, Gangnam-gu, Seoul, 135-710, Korea; 2 Samsung Biomedical Research Institute, 50 Irwon-dong, Gangnam-gu, Seoul, 135-710, Korea; 3 Department of Ophthalmology, Seoul National University Hospital, 101 Daehak-ro, Jongno-gu, Seoul 110-744, Korea; 4 Laboratory of Ocular Regenerative Medicine and Immunology, Biomedical Research Institute, Seoul National University Hospital, 101 Daehak-ro, Jongno-gu, Seoul 110-744, Korea

## Abstract

Choroidal neovascularization (CNV) is the hallmark of wet age-related macular degeneration (AMD), one of the leading causes of blindness in the elderly. Although the pathogenesis of CNV is not clear, a number of studies show that ocular-infiltrating macrophages and inflammation play a critical role in the development of CNV. TNFα-stimulated gene/protein (TSG)-6 is a multifunctional endogenous protein that has anti-inflammatory activities partly by regulating macrophage activation. Therefore, we here investigated the therapeutic potential of TSG-6 in a rat model of CNV induced by laser photocoagulation. Time course analysis showed that the expression of VEGF and pro-inflammatory cytokines in the choroid was up-regulated early after laser injury, and gradually decreased to baseline over 14 days. An intravitreal injection of TSG-6 suppressed the expression of VEGF and pro-inflammatory cytokines including CCL2, and reduced the size of CNV. Also, the number of Iba^+^ and CCR2^+^ cells including infiltrating macrophages was markedly lower in the CNV lesion of TSG-6-treated eyes. Further analysis identified CCR2^+^ CD11b^+^ CD11c^+^ cells and CCR2^+^ CD11b^-^CD11c^+^ cells as the cell populations that were increased by laser injury and reduced by TSG-6 treatment. Together, the results demonstrate that TSG-6 inhibits inflammation and CCR2^+^ monocyte recruitment into the choroid, and suppresses the development of CNV.

Age-related macular degeneration (AMD) is the most common cause of blindness in people older than 55 years in the developed world^1^,^2^,^3^,^4^. Up to 90% of visual loss in AMD is secondary to choroidal neovascularization (CNV), i.e. the growth of new blood vessels originating from the choroid through Bruch’s membrane into the sub-retinal pigment epithelium (RPE) or subretinal space^5^. The current standard treatment of CNV is repeated intravitreal injections of anti-vascular endothelial growth factor (VEGF) agents^6^,^7^,^8^. However, the treatment using anti-VEGF agents has limitations such as drug resistance, tachyphylaxis, frequent recurrences, or poor long-term visual outcome. Moreover, injections of anti-VEGF agents cannot prevent CNV development because they do not eradicate the underlying cause of CNV.

In recent years, remarkable progress has been made in our understanding of the pathogenesis of CNV. Several studies showed an association between elevated levels of CCL2 (monocyte chemoattractant protein) and CNV in human patients^9^,^10^. Also, a number of studies indicated an implication of ocular-infiltrating macrophages in the development of CNV in animal models^11^,^12^,^13^,^14^. Especially, CCR2-dependent recruitment of macrophages and their activation were critical for VEGF production and CNV formation^15^,^16^,^17^,^18^. In line with these discoveries, there have been several efforts to develop new therapies for CNV by targeting macrophage recruitment and activation^12^,^14^,^15^,^19^,^20^.

TNFα-stimulated gene/protein 6 (TSG-6) is a multi-functional, anti-inflammatory protein expressed by a variety of cells including mesenchymal stem/stromal cells (MSCs)^21^,^22^,^23^. Recent studies show that direct application of recombinant TSG-6 protein has therapeutic effects in various animal models of diseases in the eye and other tissues^24^,^25^,^26^,^27^,^28^. TSG-6 acts in part by aborting the early phase of inflammation through the modulation of NF-κB signaling in macrophages^29^,^30^,^31^.

Therefore, in this study, we investigated the therapeutic potential of TSG-6 in a rat model of CNV induced by laser photocoagulation, a well-established animal model of CNV^32^. Our results demonstrate that an intravitreal injection of recombinant TSG-6 inhibits inflammatory responses in the retina and choroid upon injury, reduces the infiltration of CCR2^+^ monocytes into the choroid, and subsequently suppresses the development of CNV.

## Results

### TSG-6 inhibits CNV development and VEGF expression in the choroid

**Figure 1:**
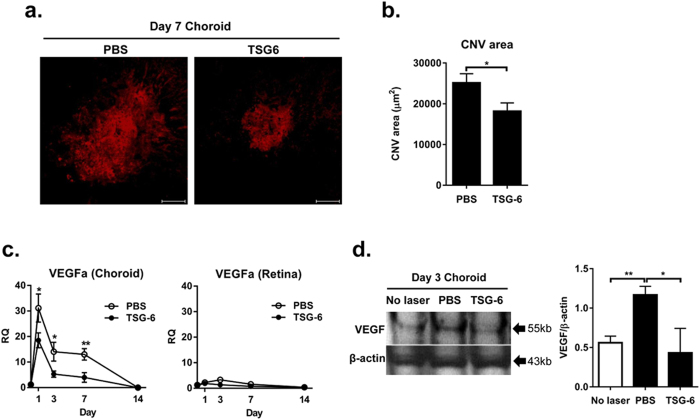
TSG-6 reduces CNV development and VEGF production in the choroid after laser injury. After laser injury to Bruch’s membrane, either recombinant TSG-6 or PBS was injected into the vitreous cavity of rat eyes. At 1, 3, 7, and 14 days after injury, the whole RPE-choroids and retinas were separated and subjected to assays. **(a)** Representative photographs of the lectin staining of the whole RPE-choroid-scleral flat mounts at day 7 showed that CNV was induced by laser injury, and TSG-6 treatment reduced CNV development. Original magnification x 200. Scale bar: 100 μm. **(b)** Bar graphs showed quantification of the CNV size in TSG-6 and PBS-treated eyes. The size of CNV was significantly smaller in the TSG-6-treated rats than in the PBS-treated controls. Data are presented as mean + SEM from three independent experiments (each with six rats per group). The SEM represents the standard error of the mean values per animal (n = 18). **(c)** Real-time RT PCR showed that the mRNA level of VEGF highly increased in the RPE-choroid at day 1 after injury, and decreased to normal over 14 days. The level of VEGF transcript in the RPE-choroid was significantly lower in TSG-6-treated eyes than in PBS-treated controls at days 1, 3, and 7. However, the expression of VEGF was not either increased in the retina by laser or affected by TSG-6 treatment. Data represent mean ±/+ SD from three independent experiments, each with six rats per group. RQ means a ratio of mRNA levels relative to those in normal eyes. **(d)** Western blotting of the RPE-choroidal tissues at day 3 and subsequent densitometry demonstrated that the amount of VEGF protein induced by injury was significantly decreased by TSG-6 treatment. Data are shown as mean band density normalized relative to β-actin + SD, and represent three independent experiments, each with six rats per group. *p < 0.05, **p < 0.01.

To evaluate clinical effects of TSG-6 on CNV development, we intravitreally injected either recombinant TSG-6 (400 ng/2 μl phosphate buffered solution; PBS) or the same volume of PBS in rats right after laser photocoagulation to Bruch’s membrane. At 1, 3, 7, and 14 days after laser injury, the whole RPE-choroidal and retinal tissues were separated and subjected to assays. We found that the area of CNV as analyzed by isolectin B4-staining in the RPE-choroid-sclera flat mounts was significantly smaller at day 7 in the TSG-6-treated rats, compared to the PBS-treated controls (Fig. 1a,b). Since the development of CNV depends on local production of VEGF^17^,^33^, we further evaluated the mRNA and protein levels of VEGF in the retina and RPE-choroid. Real-time RT PCR showed that the expression of VEGF was highly up-regulated in the RPE-choroid at day 1 after injury, and decreased thereafter (Fig. 1c). TSG-6 treatment significantly reduced the level of VEGF transcript in the RPE-choroid at days 1, 3, and 7 (Fig. 1c). Similarly, western blotting of the RPE-choroidal tissue at day 3 showed that the amount of VEGF protein was markedly less in TSG-6-treated eyes than in PBS-treated eyes (Fig. 1d). By contrast, the expression of VEGF was not induced in the retina by laser photocoagulation (Fig. 1c).

### TSG-6 reduces the levels of pro-inflammatory cytokines in the choroid and retina

**Figure 2:**
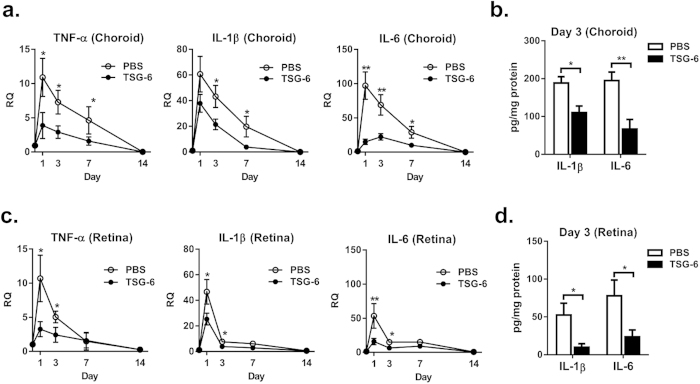
TSG-6 suppresses inflammatory responses in the choroid and retina after laser injury. **(a)** Real-time RT PCR of the RPE-choroidal tissue revealed that mRNA levels of TNF-α, IL-1β, and IL-6 were highly increased at day 1 after laser injury, and gradually decreased to baseline over 14 days. The levels of TNF-α, IL-1β, and IL-6 in the RPE-choroid were significantly lower in TSG-6-treated eyes at days 1, 3, and 7, compared to PBS-treated controls. **(b)** ELISA confirmed that the protein levels of IL-1β and IL-6 in the RPE-choroid were significantly reduced by TSG-6 treatment. **(c)** Real-time RT PCR of the retinal tissue showed that the mRNA levels of TNF-α, IL-1β, and IL-6 increased at day 1 after laser injury, and rapidly normalized until 7 days. The levels of TNF-α, IL-1β, and IL-6 transcripts in the retina were significantly lower in TSG-6-treated eyes at days 1 and 3. **(d)** Additional assay with ELISA confirmed that the levels of IL-1β and IL-6 proteins in the retina were significantly lower in TSG-6-treated group. Data are presented as mean±/+SD from three independent experiments. Each individual experiment included six rats per group at each time point. RQ means a ratio of mRNA levels relative to those in normal eyes. *p < 0.05, **p < 0.01.

We next evaluated the effects of TSG-6 on inflammatory responses in the RPE-choroid and retina after laser injury. Time course analysis revealed that the transcript levels of TNF-α, IL-1β, and IL-6 were highly increased in both RPE-choroid and retina at day 1 after injury, indicating that laser photocoagulation markedly induced inflammatory responses in the eye (Fig. 2a,c). The levels of pro-inflammatory cytokines were decreased to baseline over 14 days. An intravitreal injection of TSG-6 significantly reduced the transcript levels of TNF-α, IL-1β, and IL-6 in the RPE-choroid at days 1, 3, and 7, and in the retina at days 1 and 3 (Fig. 2a,c). Similar results were obtained with ELISA. The protein levels of IL-1β and IL-6 at day 3 were significantly lower in the RPE-choroid and retina of TSG-6-treated eyes compared to PBS-treated controls (Fig. 2b,d).

The data, therefore, indicate that an intraocular injection of TSG-6 suppresses the choroidal and retinal inflammation caused by laser photocoagulation.

### TSG-6 decreases infiltration of CCR2^+^ monocytes in the choroid

**Figure 3:**
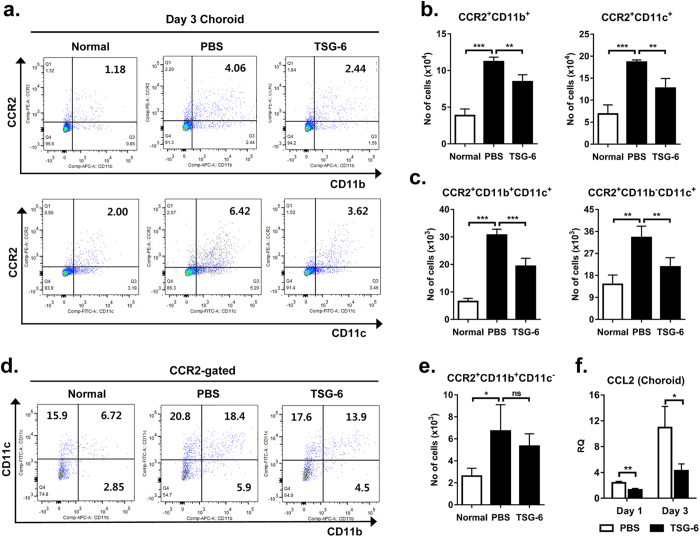
TSG-6 decreases CCR2^+^ monocyte infiltration and CCL2 production in the choroid. **(a, b)** Flow cytometric analysis showed that the number of CCR2^+^ CD11b^+^ cells and CCR2^+^ CD11c^+^ cells was highly increased in the RPE-choroid at day 3 after laser injury, and significantly reduced by an intravitreal injection of TSG-6. **(c-e)** Further analysis after gating on CCR2 revealed that the number of CCR2^+^ CD11b^+^ CD11c^+^ cells and CCR2^+^ CD11b^−^CD11c^+^ cells in the RPE-choroid was significantly decreased by TSG-6 treatment. However, the number of CCR2^+^ CD11b^+^ CD11c^−^ cells was not altered by laser injury or TSG-6 treatment. **(f)** Real-time RT PCR showed that the expression of CCL2 in the RPE-choroid was significantly suppressed by TSG-6 treatment. n = 4 in each group. Data are presented as mean + SEM. *p < 0.05, **p < 0.01, ***p < 0.001, ns: not significant.

Monocytes infiltrating the injury site are known as a major source of inflammatory cytokines and angiogenic factors such as VEGF upon laser photocoagulation, and therefore, are critical for CNV development^11^,^12^,^13^,^14^,^15^,^16^,^17^. From the previous results (Figs 1,2), we observed that an intraocular injection of TSG-6 suppressed VEGF expression and local inflammation in the laser-injured RPE-choroid. Based on these observations, we hypothesized that TSG-6 might affect the infiltration of inflammatory monocytes in the choroid after laser injury. To test the hypothesis, we analyzed the RPE-choroid and retina for CCR2, CD11b, and CD11c-expressing cells at day 3 after laser photocoagulation. We here evaluated CCR2 expression because CCR2 is highly expressed by migrating inflammatory monocytes, but not by resident microglia^34^,^35^,^36^. We used CD11b and CD11c as markers for myeloid cells or monocytes: macrophages and dendritic cells. Flow cytometric analysis revealed that the number of CCR2^+^ CD11b^+^ cells and CCR2^+^ CD11c^+^ cells, indicating migrating monocytes, was markedly increased in the RPE-choroid by laser injury, and significantly reduced by an intravitreal injection of TSG-6 (Fig. 3a,b). Further analysis after gating on CCR2 showed that TSG-6 treatment significantly decreased the number of CCR2^+^ CD11b^+^ CD11c^+^ cells and CCR2^+^ CD11b^-^CD11c^+^ cells in the RPE-choroid (Fig. 3c,d); however the number of CCR2^+^ CD11b^+^ CD11c^-^ cells was not altered by TSG-6 (Fig. 3d,e). In contrast, the number of CCR2^+^ cells, either CD11b^+^ or CD11c^+^ , was not increased in the retina at day 3 or changed by TSG-6 (Fig. S1), suggesting that monocytes did not infiltrate the retina or that monocyte infiltration did not persist until day 3.

The chemokine CCL2 is known to facilitate the recruitment of CCR2^+^ monocytes to lesion sites outside and within the CNS^37^. Also in the choroid, CCL2-CCR2 signaling is involved in the recruitment of pathogenic monocytes after laser injury^11^,^15^,^17^. Thus, we next assayed for the level of CCL2. The expression of CCL2 was markedly increased in the RPE-choroid after laser injury, and TSG-6 treatment significantly lowered the level of CCL2 in the RPE-choroid, compared to PBS treatment (Fig. 3f).

**Figure 4:**
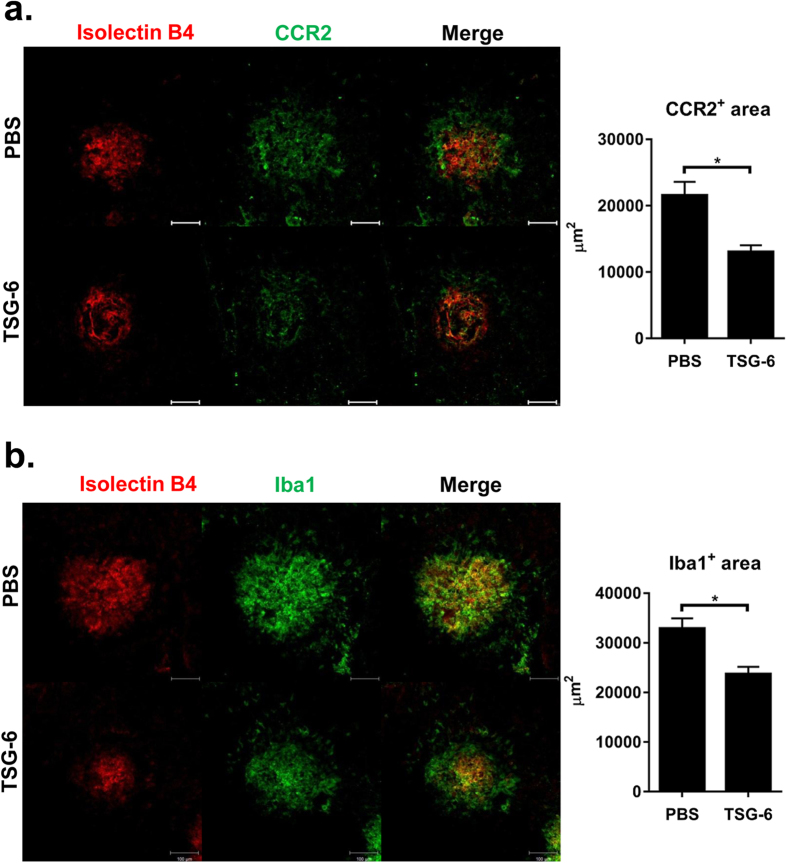
TSG-6 inhibits CCR2^+^ monocyte recruitment to CNV lesion. **(a)** Immunohistochemical staining of the RPE-choroid-scleral flat mounts at day 3 showed a massive infiltration of CCR2^+^ cells in the area of CNV after injury. There was significantly less infiltration of CCR2^+^ cells in TSG-6-treated eyes. **(b)** Similarly, Iba^+^ cells largely infiltrated the CNV lesion, and TSG-6 treatment significantly reduced Iba^+^ cell infiltration. Original magnification × 200. Scale bar: 100 μm. Photographs shown are representative of two independent experiments (each with four eyes per group), and data are presented as mean + SEM. *p < 0.05.

In addition, we performed histological assays to localize CCR2^+^ monocytes infiltrating the choroid upon laser injury. Immunohistochemical staining of the RPE-choroid-scleral flat mounts revealed large infiltration of CCR2^+^ cells in the CNV lesion at day 3 after injury (Fig. 4a). Consistent with flow cytometric results, there were significantly fewer CCR2^+^ cells in TSG-6-treated eyes (Fig. 4a). Further assay showed that a large number of Iba1^+^ cells indicating microglia and monocytes were accumulated in the lesion of CNV (Fig. 4b), reflecting the activation of microglia and infiltration of monocytes. TSG-6 treatment significantly decreased the number of Iba1^+^ cells in the injury site (Fig. 4b).

Together, the results suggest that an intravitreal injection of TSG-6 reduces CCL2 production in the choroid after laser injury, and therefore suppresses the recruitment of migratory monocytes (CCR2^+^ CD11b^+^ CD11c^+^ cells and CCR2^+^ CD11b^-^CD11c^+^ cells) to the site of injury.

## Discussion

Cells of the monocyte-macrophage lineage are key components of diverse pathological conditions^38^,^39^. To sustain macrophage infiltration at sites of injury, the recruitment of circulating monocytes through chemokines is essential, and these chemokines are produced by stromal cells and resident macrophages activated by injury^40^. Several lines of evidence indicate that ocular infiltrating macrophages play a key role in the development of CNV^11^,^12^,^13^,^14^, and CCL2-CCR2 signaling is important for the recruitment of circulating monocytes to the lesion of injury^15^,^16^,^17^,^18^. Therefore, the inhibition of macrophage activation and recruitment are potential therapeutic strategies for CNV treatment.

One strategy is to block CCL2-CCR2 signaling by antibodies or antagonists as previously reported in several studies^13^,^14^,^20^. For instance, Xie *et al.* reported that an intravitreal injection of small molecule antagonist of CCR2 was effective in decreasing macrophage infiltration, VEGF production, and CNV area in a mouse model of laser-induced CNV^20^.

Another inviting strategy for therapy is to suppress early inflammatory responses, thereby inhibiting the amplification of the pro-inflammatory signals including cytokines and chemokines such as CCL2 produced by resident macrophages^41^. One candidate molecule for this strategy is TSG-6. TSG-6 is a 30 kDa glycoprotein that has anti-inflammatory effects in a number of animal models^21^,^22^,^23^,^24^,^25^,^26^,^27^,^28^. Of special interest is the notion that TSG-6, either directly or through a complex with hyaluronan, binds to CD44 on resident macrophages in a manner that decreases TLR/NF-κB signaling and modulates the initial phase of the inflammatory response^29^,^30^,^31^. Consistent with this notion, we here observed that an intravitreal injection of TSG-6 repressed inflammation in the retina and choroid by inhibiting the production of pro-inflammatory cytokines including TNF-α, IL-1β, IL-6, and CCL2 in early phase after laser injury. Subsequently, the recruitment of CCR2^+^ monocytes to the site of injury was inhibited. VEGF production and CNV development were markedly suppressed.

One of the critical observations here was that TSG-6 decreased the infiltration of CCR2^+^ CD11b^+^ CD11c^+^ cells and CCR2^+^ CD11b^-^CD11c^+^ cells in the injury site. CCR2 is expressed by migrating cells, not by resident cells^34^,^35^,^36^. CD11b and CD11c are expressed by myeloid cells or monocytes: macrophages and dendritic cells (DCs). Although the role of infiltrating macrophages in CNV development is well-documented^11^,^12^,^13^,^14^, the contribution of infiltrating DCs to CNV pathogenesis is not yet clear. One study by Krause *et al.* showed that while the VEGF content per CD11b^+^ phagocytes markedly increased, VEGF content per CD11b^-^CD11c^+^ DCs was unchanged after laser injury, arguing against a role of DCs in CNV development^17^. Given the finding by Krause *et al.*^17^, the decrease in CCR2^+^ CD11b^-^CD11c^+^ cells (presumably DCs) in our study might be the result of decreased production of CCL2 after TSG-6 treatment, and may not directly mediate the effects of TSG-6 on CNV development. However, because of a remarkable plasticity of macrophages/DCs and ambiguity in surface markers discriminating macrophages and DCs^38^, it is difficult to clearly define the identity of the cells involved in the CNV development. Therefore, further studies would be necessary to thoroughly identify the cell types pathogenic for CNV and relative role(s) of DCs and macrophages in the pathogenesis of CNV and AMD.

We here tested the effects of a single, maximal dose of TSG-6 according to the solubility limit in an experimental CNV model. It would be necessary to compare the effects of repeated injections of TSG-6 or to determine the dose-effect relationship in the same model.

One caution of this study is that reduction in the isolectin B4-labeled area might not solely reflect reduced CNV, because microglial cells as well as vascular endothelial cells are labeled with isolectin B4. However, microglial cells can be distinguished from endothelial cells in that microglial cells are superficially located relative to CNV, and have dendritic morphology without tube formation. Therefore, we excluded such segregated areas surrounding CNV in the measurement of CNV size. Hence, it is likely that reduced isolectin B4-labeled area observed in our study mostly represented a reduction in the CNV size^42^.

Another caution is that there are CCR2-independent cells producing VEGF in CNV^17^. In addition, CCL2-CCR2 signaling was shown to be also involved in the recruitment of neuroprotective and anti-inflammatory macrophages^37^,^43^ as well as inflammatory monocytes. Therefore, these studies suggest a potential limitation in the use of CCL2-CCR2 inhibitors for treatment of CNV and AMD.

In summary, our results demonstrate that an intravitreal injection of recombinant TSG-6 protein inhibits early inflammatory response in the retina and choroid after injury, reduces CCR2^+^ monocyte recruitment to the choroid, and suppresses VEGF production and CNV development in the choroid. These findings support the notion that targeting inflammation may be beneficial for treatment of patients with CNV and AMD, and have implications for the design of combination therapies. Furthermore, the results provide a rationale to further explore TSG-6 as a novel therapy for patients with CNV and other intraocular inflammatory disorders.

## Methods

### Animals and laser-induced CNV model

The experimental protocol was approved by the Institutional Animal Care and Use Committee of Samsung Medical Center. Animals were treated in strict accordance with the ARVO statement for the use of animals in ophthalmic and vision research. Brown Norway rats (Japan SLC, Hamamatsu, Japan) weighing 200–250 g were used in all experiments. Laser photocoagulation was performed as previously described^44^,^45^. Briefly, under anesthesia with an intraperitoneal injection of zolazepam-tiletamine (Zoletil®, Virbac, Carros, France), the pupils were dilated, and six laser spots (532 nm wavelength, 200 mW power, 100 ms duration, 75 μm spot size) were applied to Bruch’s membrane around the optic nerve head using slit lamp delivery system. The eyes with the burns that generated a bubble implying the rupture of the Bruch membrane were included, and the eyes that had spot(s) containing hemorrhage were excluded from further analysis. Immediately after laser photocoagulation, either recombinant TSG-6 (400 ng/2 μl PBS; R&D Systems, Minneapolis, MN) or the same volume of PBS was injected intravitreally using a 33 gauge needle (Hamilton, Reno, NV).

### Measurement of CNV

To measure the size of CNV^44^,^45^, the eyes were extracted and fixed in 4% paraformaldehyde. Then, the RPE-choroid-scleral cups were isolated and incubated in 5% bovine serum albumin (BSA)/PBS for 4 h. The TRITC–conjugated *Bandeiraea simplicifolia* (BS) isolectin B4 (0.2 mg/mL; Sigma-Aldrich, St. Louis, MO) in Pblec buffer (PBS, pH 6.8, 1 mM CaCl_2_, 1 mM MgCl_2_, 1 mM MnCl_2_, 1% Triton X-100) was applied to the eyecups at 4 °C overnight. After thorough washing with PBS, the RPE-choroid-sclera tissues were flat-mounted, and examined under a confocal microscope (LSM700, Carl Zeiss MicroImaging GmbH, Jena, Germany). The images of laser spots were observed with a wide pinhole allowing a large depth of field. The area of CNV was measured at a depth where the largest dimension of CNV was observed among the images using Image J program (National Institute of Health, Bethesda, MD, USA) by an ophthalmologist who was not aware of the treatment of the rats as previously described^32^,^46^.

### Immunofluorescence staining

Together with TRITC–conjugated BS isolectin B4, mouse monocloncal anti-Iba1 antibody (1:50, ab15690, Abcam, Cambridge, MA) or rabbit monocloncal anti-CCR2 antibody (1:250, ab32144, Abcam) was applied to the eyecups at 4 °C overnight. After washing with PBS, secondary antibody (FITC-conjugated anti-mouse or anti-rabbit IgG, 1:500) in 2% BSA/PBS was applied at room temperature for 1 h. After washing, the RPE-choroid-scleral tissues were flat-mounted, and examined under a confocal microscope (LSM700). The images of laser spots were captured, and the area of Iba1- or CCR2-stained area was measured using Image J program by an ophthalmologist in a blinded manner.

### Real-time RT-PCR

The whole retinas or RPE-choroids were separated all the way to the ora serrata in each eye, and cut into small pieces with microscissors. Then the tissues were lysed in RNA isolation reagent (RNA Bee, Tel-Test Inc., Friendswood, TX), and sonicated with a probe sonicator (Ultrasonic Processor, Cole Parmer Instruments, Vernon Hills, IL). Total RNA was extracted using an RNeasy Mini kit (Qiagen, Valencia, CA), and the double-stranded cDNA was synthesized from the same amount of RNA per eye of each group by reverse transcription (High Capacity RNA-to-cDNA Kit; Applied Biosystems, Carlsbad, CA). Real-time amplification was performed with a TaqMan Universal PCR Master Mix (Applied Biosystems). A rat GAPDH was used for normalization of gene expression. The PCR probe sets of rat GAPDH, VEGF, TNF-α, IL-1β, IL-6, and CCL2 were purchased from Applied Biosystems (Taqman Gene Expression Assay kits).

### Western blot

For protein extraction, the tissues were minced into small pieces with microscissors and lysed in PRO-PREP^TM^ Protein Extraction Solution (Intron Biotechnology, Seongnam, Korea). The samples were then sonicated on ice with an ultrasound sonicator (Ultrasonic Processor), and the supernatant was collected after centrifugation at 12,000 rpm for 20 min. A total of 30 μg protein was fractionated by SDS-PAGE on 10% bis-tris gel (Invitrogen, Carlsbad, CA), transferred to nitrocellulose membrane (Invitrogen), and blotted with antibodies against rat VEGF (Millopore, Temecula, CA) or β–actin (Santa Cruz, Delaware CA).

### Elisa

Clear lysates of protein from the retinas or RPE-choroids were prepared as described above, and assayed for the concentrations of IL-1β and IL-6 by ELISA (Rat Duoset kit, R&D Systems) according to the manufacturer’s protocol.

### Flow Cytometry

The retinas or RPE-choroids were cut into small pieces with microscissors, and incubated in collagenase IV (2 mg/ml) at 37 °C for 1 h under continuous agitation. The tissues were filtered through a nylon mesh, and the cells were collected after centrifugation at 18,000 rpm for 3 min. After washing with RPMI 1640 media (Welgen, Daegu, Korea), the cells were incubated at 4 °C for 30 min with PE-conjugated anti-rat CCR2 (R&D Systems), APC-conjugated anti-rat anti-CD11b (BD BioSciences, San Diego, CA), and FITC-conjugated anti-CD11c antibodies (Bio-Rad, Hercules, CA ). Also, the cells were stained with propidium iodide for gating of live (PI^−^) cells, and the debris was excluded from analysis based on forward and side scatter signals. A total of 10,000 viable cells per eye were analyzed for fluorescence using a FACSCanto flow cytometer (BD BioSciences). Data were analyzed using Flowjo program (Tree Star, Inc., Ashland, OR).

### Statistical analysis

The data are expressed as the mean ± SEM or SD. Comparisons of two values between the groups were made using the Mann-Whitney *U*-test, and comparisons of more than two means were made using a one-way ANOVA (Prism®, GraphPad Software, Inc., La Jolla, CA). Differences were considered significant at p < 0.05.

## Additional Information

**How to cite this article**: Kim S. J. *et al.* Intravitreal TSG-6 suppresses laser-induced choroidal neovascularization by inhibiting CCR2^+^ monocyte recruitment. *Sci. Rep.* **5**, 11872; doi: 10.1038/srep11872 (2015).

## Supplementary Material

Supplementary Information
