# Supplementary material for: Intravitreal TSG-6 suppresses laser-induced choroidal neovascularization by inhibiting CCR2+ monocyte recruitment
Source: Sci Rep. 2015 Jul 7;5:11872. doi: 10.1038/srep11872 (PMC4493567; doi:10.1038/srep11872)

Title: Intravitreal TSG-6 suppresses laser-induced choroidal neovascularization by inhibiting $CCR2^+$ monocyte recruitment.

Short title: Effect of TSG-6 in choroidal neovascularization

Authors: Sang Jin Kim,[1, 2 *] Hyun Ju Lee,[3, 4] Ji-Hyun Yun,[2] Jung Hwa Ko,[3, 4] Da Ye Choi,[1] Joo Youn Oh[3, 4 *]

***SJK and JYO equally contributed to the work.

Affiliations:

[1] Department of Ophthalmology, Samsung Medical Center, Sungkyunkwan University School of Medicine, 50 Irwon-dong, Gangnam-gu, Seoul, 135-710, Korea.

[2] Samsung Biomedical Research Institute, 50 Irwon-dong, Gangnam-gu, Seoul, 135-710, Korea.

[3] Department of Ophthalmology, Seoul National University Hospital, 101 Daehak-ro, Jongno-gu, Seoul 110-744, Korea.

[4] Laboratory of Ocular Regenerative Medicine and Immunology, Biomedical Research Institute, Seoul National University Hospital, 101 Daehak-ro, Jongno-gu, Seoul 110-744, Korea.

The authors declare that no competing financial or personal interests exist.

Supplementary Figure 1. Flow cytometric analysis of retinal cells at day 3 after laser photocoagulation to Bruch's membrane.

TSG-6 treatment did not affect the percentage of $CCR2^{+}CD11b^{+}$ or $CCR2^{+}CD11c^{+}$ cells in the retina.

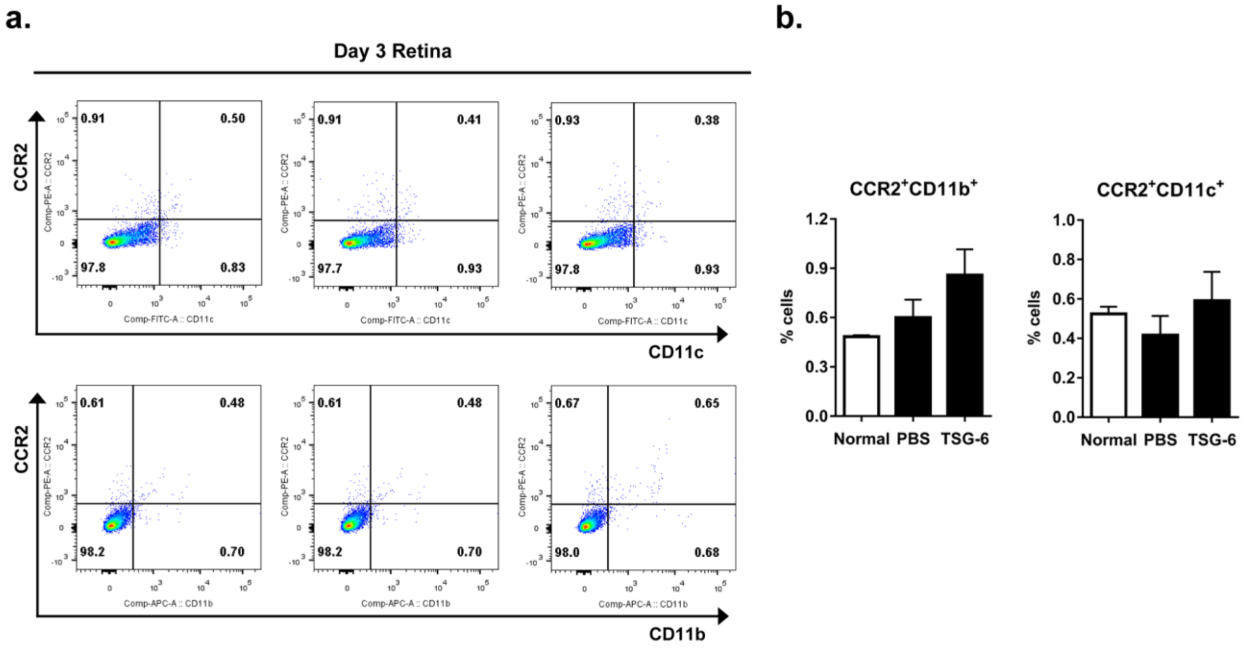

Supplement: Supplementary Information [file srep11872-s1.pdf]
